# Efficacy of lenalidomide in association with cyclophosphamide and dexamethasone in multiple myeloma patient with bilateral retro-orbital localisation

**DOI:** 10.3332/ecancer.2013.331

**Published:** 2013-07-10

**Authors:** S Felici, N Villivà, G Balsamo, A Andriani

**Affiliations:** 1 Haematology Unit, Nuovo Regina Margherita, Hospital, 00153 Rome, Italy; 2 Histopathology Complex Unit, Santo Spirito Hospital, 00193 Rome, Italy

**Keywords:** multiple myeloma, extramedullary disease, ocular involvement, lenalidomide

## Abstract

Extramedullary localisation is an uncommon manifestation in multiple myeloma (MM). Ocular involvement is rare. Here, we describe a relapse of MM with bilateral retro-orbital localisation without any bone involvement with good and rapid response to therapy with lenalidomide, dexamethasone, and cyclophosphamide.

## Introduction

Multiple myeloma (MM) accounts for 1% of malignant diseases and for 10% of haematologic malignancies [1].

It is a clonal B cell tumour characterised by monoclonal protein production and by the slow proliferation of plasma cells, mainly in bone marrow.

Marrow infiltration by plasma cells, monoclonal protein production, and the reaction of the bone marrow microenvironment are the causes of lytic bone lesions, anaemia (with or without thrombocytopenia and leucopenia), loss of normal immune response, renal failure, and hypercalcaemia.

According to the International Myeloma Working Group, to make a diagnosis of MM it is necessary to demonstrate the presence of at least 10% plasma cells on bone marrow examination or on extramedullar tissue biopsy, as well as monoclonal protein (in serum or urine) and organ damage [2].

The acronym CRAB defines organ damage: hypercalcaemia, renal failure, anaemia, bony lesions [3–5].

Solitary plasmacytoma of the bone represents 3%–5% of plasma cell dyscrasias. In addition to finding a tumour composed of light chain restricted plasma cells on biopsy, in this case it is necessary to demonstrate the presence of a normal bone marrow and the absence of other skeletal lesions [through magnetic resonance imaging (MRI), positron emission tomography–computed tomography (PET–CT) scans], and the presence of monoclonal protein in serum and urine. Almost 50% of patients with plasmacytoma can develop an MM [6]. 

Extramedullary plasmacytoma can also occur in soft tissue. It is more frequent in the tonsils, nasopharynx, gastrointestinal tract, and paranasal sinuses. To identify it correctly, the absence of bony lesions and plasma cell infiltration in bone marrow must be demonstrated. Monoclonal protein can be present. Radiotherapy is the treatment of choice associated—if necessary—with surgical resection [7].

Extramedullary disease (EMD) is a clinical manifestation of myeloma. Bladè *et al *reported that the incidence of EMD in newly diagnosed myeloma ranges from 7% to 18%; 6% to 20% of patients can develop EMD at a later stage of the disease [8].

EMD can arise from the destruction of cortical bone at the starting point of a tumour or from haematogenous metastatic spread [8]. Local growth after the destruction of cortical bone is the most frequent. Haematogenous spread is more frequent in the skin, liver, kidneys, or in the nervous system.

Ophthalmic involvement is rare in MM and can damage any structure of the eye [4, 9].

Here, we describe the case of a relapsed MM patient with bilateral retro-orbital localisation without any bone involvement who was treated with lenalidomide, dexamethasone and cyclophosphamide.

## Case report

In February 2010, a 73-year-old male patient arrived at our institution because of anaemia, which was diagnosed in January 2010 after laboratory tests were performed to investigate the origin of thoracic pain. His haemoglobin levels were 8.5 gr/dl, and the other blood tests showed moderate renal failure and the presence of a monoclonal component IgG (K). The skeletal X-ray was negative with regard to the presence of osteolysis, and the bone marrow biopsy showed plasma cell infiltration of about 50%.

Symptomatic MM was diagnosed, and a treatment with bortezomib (Velcade) biweekly (days 1, 4, 8, 11, 22, 25, 28, 32) Melphalan and Prednisone from day 1 to day 4, every 35 days (VMP) was started.

The chemotherapy, combined with diuretic treatment and erythropoietin support (epoetin alfa 40,000 units subcutaneous weekly), was completed after four cycles in August 2010. The patient achieved a subjective improvement with a documented recovery of the renal function as well as an improvement of anaemia and the disappearance of dorsal pain.

Even though the partial remission was documented by the reduction of the monoclonal component, to 1 gr/dl, the patient presented neurological symptoms characterised by difficulty in walking and diplopia due to the paralysis of the oculomotor nerve. Before the fifth cycle of VMP, he was admitted to the Department of Neurology, where a bortezomib-related neuropathy was diagnosed [52].

During hospitalisation, the patient obtained a partial recovery of ambulation and therefore a specific treatment for MM—only including melphalan and prednisone—was restarted after discharge and continued for another four cycles. In November 2010, distal oedemas associated with hyponatraemia and hypoalbuminaemia appeared, and treatment with diuretics and albumin was started. The IgG blood levels became <1 g/dl, and the bone marrow aspiration showed a reduction of bone-marrow plasma cells up to 1%–2% of all cellularity as well as red cells progenitor hyperplasia. Periumbilical fat aspiration was negative to Congo red stain.

Maintenance with monthly dexamethasone and zoledronic acid was performed.

**Figure 1:**
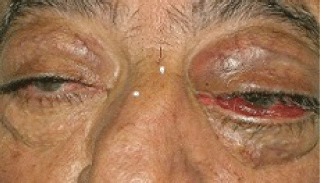
Bilateral exophthalmos (left > right), periorbital oedema, conjunctival hyperaemia.

**Figure 2:**
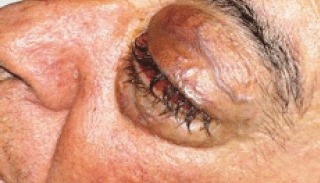
Detail of left eye.

In August 2011, the patient came to our Day Hospital with bilateral exophthalmos (left > right), periorbital oedema, conjunctival hyperaemia, diplopia, continuous pain, and decreased vision (Figures 1 and 2).

An MRI scan of the central nervous system showed inspissations of the extrinsic ocular muscles and especially of the left medial rectus muscle. A biopsy was proposed but could not be performed due to technical problems.

At this point, to reduce the continuous pain and the decreased vision, we started treatment with cyclophosphamide (500 mg/w i.v. for three consecutive weeks and one of suspension), lenalidomide [10 mg/daily orally days 1–21 every 28 days], and dexamethasone (40 mg intravenously, days 1–2, 8–9, 15–16) (L-CD) [6, 7].

**Figure 3:**
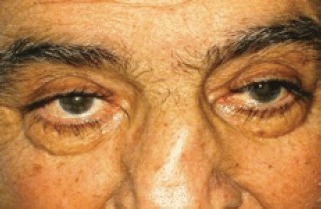
The exophthalmos, periorbital oedema, conjunctival hyperaemia disappeared after first cycle of therapy.

**Figure 4:**
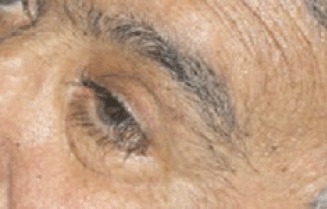
Detail of left eye after first cycle of therapy.

After the first cycle, the exophthalmos, periorbital oedema, conjunctival hyperaemia, diplopia, and continuous pain disappeared (Figures 3 and 4).

Treatment was modified at the third cycle for better patient compliance, and cyclophosphamide (50 mg orally days 1–21) in combination with lenalidomide was prescribed for three more cycles.

Until April 2012, the patient continued treatment and maintained complete response but unfortunately, in May 2012, the patient died due to cardiac arrest.

## Discussion

MM may cause ocular pathologies by direct infiltration or as an EMD. This results in the displacement or compression of tissues, by causing hyperviscosity syndrome and by immunoglobulin light chain deposition in ocular tissues. Rarely, ocular findings may be the first manifestation of the disease; more frequently they may occur as one of the extramedullary manifestations of the disease and as the sign of insufficient chemotherapy or a relapse [10].

In the retina, cotton-wool spots, nerve fibre dropout, haemorrhages, and vascular abnormalities (including dilatation, tortuosity, and microaneurysms) can develop secondary to the associated serum hyperviscosity. Optic nerve, oculomotor nerve and lacrimal gland infiltration by myeloma cells have been reported. Iridescent crystalline or copper deposits in the conjunctiva and cornea, ciliary body cysts, and cranial nerve compression causing palsies have also been described [9].

Orbital involvement occurs more frequently than intraocular involvement and is typically diagnosed through CT [10, 11].

In the past, several authors reported the occurrence of ocular manifestations in MM [12–30].

In 2011, Chin *et al *performed a revue of the literature, presenting three cases of ocular lesions in MM [9]: Sanders *et al *in 1967 [13], reported the occurrence of ocular lesions in 12/15 eyes (80%) examined in autopsy studies and showed that these localisations are more frequent than clinically noted.

In 1972, Rodman and Font described 30 cases with ocular lesions associated with other signs of systemic disease [14].

Burkat *et al *in 2009 reviewed 52 cases reported in 41 articles; of these, 88% presented a unilateral lesion, 81% proptosis, 23% decreased vision, 23% diplopia, 21% swelling, and 13% ptosis [28].

If ocular lesions are suspected, a CT (or MRI or PET-CT) scan must be performed as well as, if possible, a biopsy of the lesion to demonstrate the presence of plasma cells CD 56, CD 38 and CD 138 positive, CD 20 negative.

The treatment of MM evolved substantially over the last decade, most notably with the introduction of highly effective novel agents (thalidomide, lenalidomide and bortezomib) the use of which resulted in considerable improvements in the outcome [31–35].

Lenalidomide has immunomodulatory effects, anti-inflammatory, antiangiogenic and proapoptotic activities; lenalidomide has direct effects on tumour cells, the tumour microenvironment, cytokine responses, production of growth factors, and T cell activation [40–43].

Lenalidomide combined with dexamethasone is an effective treatment for patients with relapsed/refractory MM and is associated with increased response rates and prolonged progression-free survival and overall survival (OS) compared with dexamethasone alone [36, 37, 49, 50]. The Len + Dex regimen was effective regardless of the type of prior therapy received [44, 45]. Moreover Len + Dex was found to be effective when given at first relapse [46]. Continued treatment led to greater depth of response and improved survival outcomes [47, 48]. The addition of cyclophosphamide appeared to enhance the efficacy of Len + Dex, suggesting that such combination therapy may be used more in clinical practice [38, 39]. Furthermore, lenalidomide and dexamethasone are effective for EMD in refractory or relapsed myeloma (overall response rate of 61.1% with compete disappearance in 44.1%) [51].

## Conclusion

Our patient successfully went through a first-line therapy based on bortezomib, melphalan and dexamethasone (four cycles), resulting in a partial response. However, this treatment had to be stopped following the development of a severe neuropathy. The melphalan/prednisonebased treatment (in combination with dexamethasone and zoledronic acid) was successfully continued, resulting in a very good partial response up to four months before the relapse.

When the extramedullary relapse the percentage of bone-marrow plasma cells was <5%, with monoclonal IgG < 1 g/dl. Therefore, we decided to start a new combination of treatment, based on lenalidomide. To attain a quicker response, this treatment was supplemented with dexamethasone and cyclophosphamide.

Efficacy of new biologic treatments on extraosseous (ocular) localisations of MM is a highly debated question. To date, only a few case reports have been described.

We report this case due to the rapid response to the therapy of the retro-orbital localisation.

## Conflicts of interest

The authors declare that they have no conflicts of interest.
